# Synthesis and Characterization of TiO_2_-ZnO-MgO Mixed Oxide and Their Antibacterial Activity

**DOI:** 10.3390/ma12050698

**Published:** 2019-02-27

**Authors:** Luis M. Anaya-Esparza, Efigenia Montalvo-González, Napoleón González-Silva, María D. Méndez-Robles, Rafael Romero-Toledo, Elhadi M. Yahia, Alejandro Pérez-Larios

**Affiliations:** 1 Laboratorio Integral de Investigación en Alimentos, Tecnológico Nacional de México-Instituto Tecnológico de Tepic. Av., Tecnológico 255, Lagos del Country, Tepic 63175, Nayarit, México; l_m_ae@hotmail.com (L.M.A.-E.); efimontalvo@gmail.com (E.M.-G.); 2 Universidad de Guadalajara, Centro Universitario de los Altos. División de Ciencias Agropecuarias e Ingenierías. Carretera a Yahualica Km. 7.5, Tepatitlán de Morelos 47600, Jalisco, México; napoleon.gonzalez@cualtos.udg.mx (N.G.-S.); mdmendez@cualtos.udg.mx (M.D.M.-R.); 3 Universidad de Guanajuato, División de Ciencias Naturales y Exactas, Campus Guanajuato, Noria Alta S/N, Noria Alta, Guanajuato 36050, Guanajuato, México; Ing_Romero2009@hotmail.com; 4 Facultad de Ciencias Naturales, Universidad Autónoma de Querétaro. Avenida de las Ciencias S/N, Juriquilla, Santiago de Querétaro 76230, Querétaro, México; yahia@uaq.mx

**Keywords:** sol-gel method, mixed oxides, nanomaterials, antimicrobial activity

## Abstract

TiO_2_-ZnO-MgO mixed oxide nanomaterials (MONs) were synthetized via the sol-gel method and characterized by scanning electron microscopy (SEM) coupled with energy dispersive spectroscopy (EDS), transmission electron microscopy (TEM), nitrogen physisorption analysis, X-ray diffraction (XRD), UV-Vis diffuse reflectance spectroscopy (UV-Vis DRS), Fourier transform infrared spectroscopy (FTIR), and color (Luminosity (*L*), *a*, *b*, Chrome, hue) parameters. Furthermore, the antimicrobial activity of the MONs was tested against *Escherichia coli* (EC), *Salmonella paratyphi* (SP), *Staphylococcus aureus* (SA), and *Listeria monocytogenes* (LM). The MONs presented a semi globular-ovoid shape of ≤100 nm. Samples were classified as mesoporous materials and preserved in the TiO_2_ anatase phase, with slight changes in the color parameters of the MONs in comparison with pure TiO_2_. The MONs exhibited antimicrobial activity, and their effect on the tested bacteria was in the following order: EC > SP > SA > LM. Therefore, MONs could be used as antimicrobial agents for industrial applications.

## 1. Introduction

Mixed oxide systems have a wide range of applications, including physics, chemistry, materials science and engineering [1]. One of the most studied applications of inorganic nanoparticles is their antimicrobial activity [2]. This is due to the stability in harsh processing conditions (high pressure or temperature) exhibited by inorganic compounds (TiO_2_, ZnO, MgO and others) compared with organic compounds (organic acids, essential oils, bacteriocins and enzymes) [3].

The sol-gel method is an interesting synthesis method for preparing hybrid materials (mixed oxide systems and/or inorganic-organic systems), which involves hydrolysis and condensation reactions on the precursors [4]. Furthermore, this method has several advantages (simple synthesis process, mild reaction conditions, high homogeneity of the product, low energy cost, versatility, and moreover, complex apparatus is not needed), which enable its use in a wide range of technological processes. Nonetheless, one of the main advantages of the sol-gel method is the possibility of synthesizing new materials with designed properties [5,6]. This method may also be used to obtain ternary mixed oxide systems containing TiO_2_ [7,8], which represents an interesting base for the synthesis of multifunctional oxide systems [6].

Titanium dioxide is a multifunctional material [9], highly employed in the food (chewing gum with mint flavor, dairy products) and pharmaceutical (sunscreens and toothpaste) industries [10,11]. However, most of the TiO_2_ uses, including antibacterial activity, are usually UV-irradiation dependent [1,2], in particular for TiO_2_ in the anatase phase (active under UV rays at a wavelength of 385 nm or shorter), which is a limiting factor for their potential applications [12]. Furthermore, it has been reported that TiO_2_ can be combined with selective elements, forming mixed oxide materials offering an effective method to enhance the physicochemical and antimicrobial properties of TiO_2_ [1,2,13].

It has been reported that TiO_2_ in the presence of UV-irradiation exhibits antimicrobial activity against *Escherichia coli*, *Salmonella typhi*, *Klebsiella pneumonia*, *Shigella flexneri* and *Staphylococcus aureus* [2,12,13,14]. Furthermore, TiO_2_ has been doped with different materials with known antimicrobial activity such as Ag [15], Nd^3+^, Zn^2+^ [2], MgO [16] and copper [12] to enhance its property. Most of the previous studies focused on the synthesis, characterization and evaluation of the antibacterial effect of the individual or bimetallic combinations of these materials [12,13,14]. However, research based on the ternary oxide system is limited, particularly for antimicrobial proposes. Juma et al. [17] synthesized and characterized a nanocomposite formed by CuO-NiO-ZnO (using the co-precipitation method) and reported that the presence of three materials impacted strongly on the overall properties of the composite. Li et al. [18] prepared a ZnO-CeO_2_-TiO_2_ composite by the combustion method for methylene blue degradation, enhancing their photocatalytic properties. Li et al. [19] prepared a ternary organometallic composite (Ag-TiO_2_-chitosan, using the inverse emulsion cross-linking reaction), which exhibited excellent antibacterial activity against *E. coli*, *P. aeruginosa* and *S. aureus*. Arandiyan and Parvari [20] reported that the sol-gel technique is an attractive and effective method for synthesizing ternary mixed systems (LaMo*_x_*V_1_O_3+δ_). In this work, we synthesized a ternary oxide system (TiO_2_-ZnO-MgO) using the sol-gel method, and characterized this system using SEM-EDS, TEM, nitrogen physisorption analysis, XRD, FTIR, UV-Vis DRS, and color attributes. In addition, their antibacterial activity against *Escherichia coli*, *Salmonella paratyphi*, *Staphylococcus aureus* and *Listeria monocytogenes* was evaluated.

## 2. Materials and Methods

### 2.1. Material Preparation

Materials (TiO_2_-ZnO-MgO) were prepared using the sol-gel method using titanium-(IV) butoxide, zinc nitrate and magnesium di-ter-butoxide as precursors (reagents obtained from Sigma-Aladrich Chemical Co., St. Louis, MO, USA), where 44 mL of ethanol and 18 mL of distilled water were mixed with different precursor amounts to obtain solids of 1%, 3% and 5% weight (wt.) of Zn and Mg (Table 1). Then, a few drops of HNO_3_ (0.1 M) were added in order to adjust the pH to 3 in the solutions. The solutions were heated under reflux at 70 °C and 44 mL of titanium(IV) butoxide were then added drop wise and maintained during 24 h under magnetic stirring until the gels were formed. The gels were dried at 100 °C for 24 h and the solids were ground. Finally, the obtained xerogels were annealed at 500 °C/5 h in static air atmosphere (heating rate of 2 °C/min). A reference pure TiO_2_ sample was prepared in the same way described above but without the addition of the precursors (Zn-Mg) [7].

### 2.2. Sample Characterization

The morphology of the samples was determined using transmission electron microscopy (TEM) (Tecnai F20 microscope, Phillips Co., Amsterdam, The Netherlands) operated at 200 kV, and by scanning electron microscopy (SEM) (Tescan, MIRA3 LMU, London, UK) at 20 kV, equipped with an energy dispersive X-ray spectroscope (EDS, XFash sve 6/30, Bruker, Berlin, Germany). 

The textural properties were determined by nitrogen adsorption-desorption with a Micromeritics (TriStar II Plus, Norcross, GA, USA). The specific surface areas were calculated by means of the Brunauer-Emmett-Teller (BET) method and the pore size distribution was obtained according to the Barret-Joyner-Halenda (BJH) method. 

The crystallinity of the samples was characterized by X-ray diffraction (XRD; Empyrean, Malvern Panalytical, Almelo, The Netherland) equipped with Cu Kα radiation (λ = 0.154 nm). The UV-Vis absorption spectra were obtained with a UV-Vis spectrophotometer (Shimadzu UV-2600, Tokyo, Japan) coupled with an integration sphere for diffuse reflectance studies. From the plot, the bang gap energy was calculated using Plank’s Equation (1).

(1) $$Eg=\frac{1239.8}{\lambda },$$

where energy (Eg) = band gap energy (eV), and wavelength (λ) = absorption peak value.

The FTIR spectrum for the material was recorded with a FTIR (Nicolet iS5, ThermoFisher Scientific, Tokyo, Japan) spectrometer using attenuated total reflectance (ATR). The spectrum was recorded at room temperature, with 24 scans and 4 cm^−1^ resolution. Samples were recorded at a wavelength from 4000 to 400 cm^−1^. 

Mixed oxide color (Luminosity (*L*), *a*, *b*, chrome and hue values) was measured using a Minolta Colorimeter (Konica Minolta CR-400, Konica Minolta Inc., Osaka, Japan). Total color difference (TCD) was determined using Equation (2), which indicates the magnitude of the color change of the powders in the presence of dopant material [21].

(2) $$\mathrm{TCD}=\sqrt{{(L-L_{0})}^{2}+(a-a_{0}{)}^{2}+(b-b_{0}{)}^{2}},$$

where $L_{0}$, $a_{0}$, $b_{0}$ are color values of undoped TiO_2_, and *L*, *a* and *b* are values of doped TiO_2_ materials.

### 2.3. Antibacterial Activity Test

The antibacterial activity of the mixed oxide system on Gram-negative (*Salmonella paratyphi* ATCC 9150 and *Escherichia coli* ATCC 8739) and Gram-positive (*Staphylococcus aureus* ATCC 33862 and *Listeria monocytogenes* ATCC 15313) bacteria was tested by agar disc diffusion assay [1]. Microbial strains were grown aerobically in Mueller-Hinton broth (21 g/L, pH 7.3 ± 0.1) for 24 h at 37 °C until the bacterial suspensions were achieved to 1 × 10^6^ CFU/mL by comparison with the 0.5 McFarland standard. The disc diffusion assay was carried out by swabbing each test strain on Muller-Hinton agar (38 g/L, pH 7.3 ± 0.1). Sterile standard filter paper discs (4 mm in diameter) were then impregnated with sterile aqueous suspensions of mixed oxide nanomaterials at 100 μg/mL concentration and placed onto the inoculated plates using sterile forceps. The standard antibiotic drug ampicillin (10 μg/mL) and sterile distilled water were used as positive and negative controls, respectively [13]. Later, the plates were incubated at 37 °C for 24 h. Finally, the zone of inhibition that formed around the discs (diameter) was measured in millimeters (mm) and recorded. The procedure described above was repeated for each treatment (T1–T5) and each bacteria.

### 2.4. Statistical Data Analysis

Color parameters (*a* coordinate, chrome and hue values) and antimicrobial activity for *Listeria monocytogenes* and *Staphylococcus aureus* data were subjected to a one-way ANOVA/Tukey test. Their variances were shown to be homogeneous (Levene’s test, *p* > 0.05) and they also presented a normal distribution (Shapiro-Wilk W test, *p* > 0.05). Color parameters such as luminosity and *b* coordinate, as well as antimicrobial activity for *Escherichia coli* and *Salmonella paratyphi* data were subjected to the independent-samples Kruskal-Wallis non-parametric test, due to the lack of homogeneity in the variances among the groups (Levene’s test, *p* < 0.05) and/or normal distribution (Shapiro-Wilk W test, *p* < 0.05) (see Table S1 in Supplementary Materials). A pairwise comparison was performed using multiple comparisons of mean ranks for all groups. All data were obtained from three independent experiments and each sample was performed in triplicate. Results were expressed as mean ± standard deviation. Data were analyzed using the Statistica software (v. 10 Statsoft^®^, Tulsa, OK, USA), with a significance level of α = 0.05.

## 3. Results and Discussion

### 3.1. Morphological Observation and EDS Analysis

**Figure 1:**
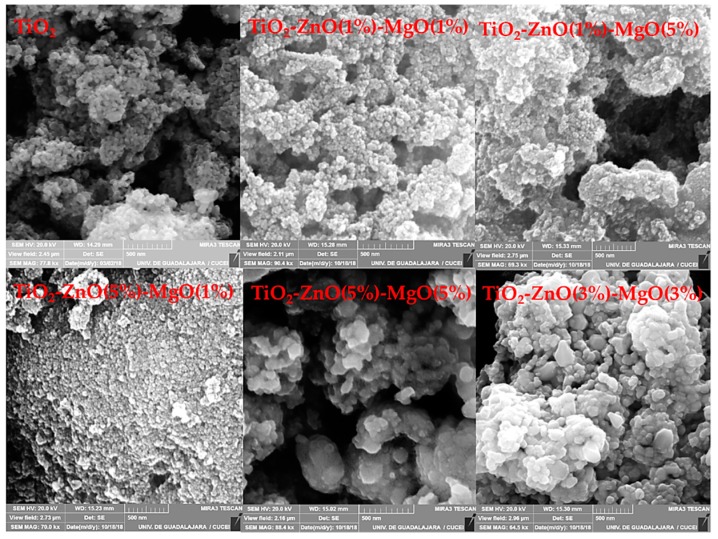
Scanning electron microscopy (SEM) images from pure TiO_2_ and mixed oxide materials.

**Figure 2:**
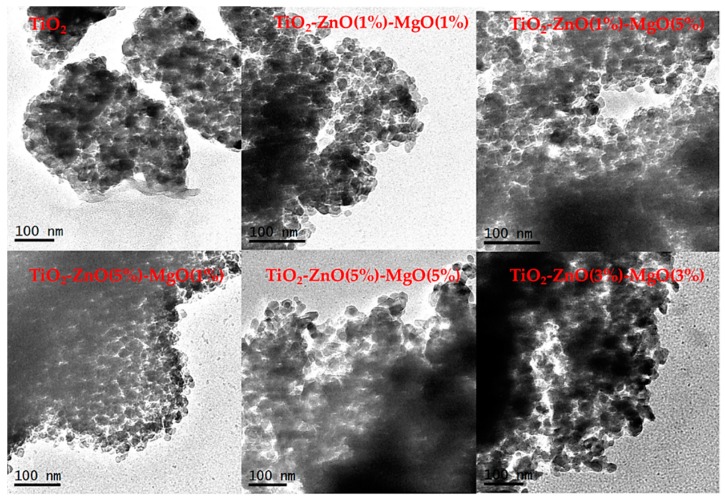
Transmission electron microscopy (TEM) images from pure TiO_2_ and mixed oxide materials.

The SEM and TEM studies of pure TiO_2_ and MONs are shown in Figure 1 and Figure 2, respectively. The images show that the materials exhibited a semi-globular form with some superficial agglomerations and sizes less than 100 nm [1]. Several nanoparticle shapes and superficial textures (including non-uniform size and superficial agglomeration) have been reported for TiO_2_, which appeared to be a normal reaction during the sol-gel method synthesis [8,9,22]. The elemental composition of the synthesized mixed oxide (TiO_2_-ZnO-MgO) nanomaterials was determined by energy dispersive X-ray analysis by SEM (Table 1). The results confirm the presence of the three metals employed in this study; furthermore, the MONs composition was mainly determined by the amounts of the initial precursors [8].

### 3.2. N_2_ Physisorption Analysis

**Figure 3:**
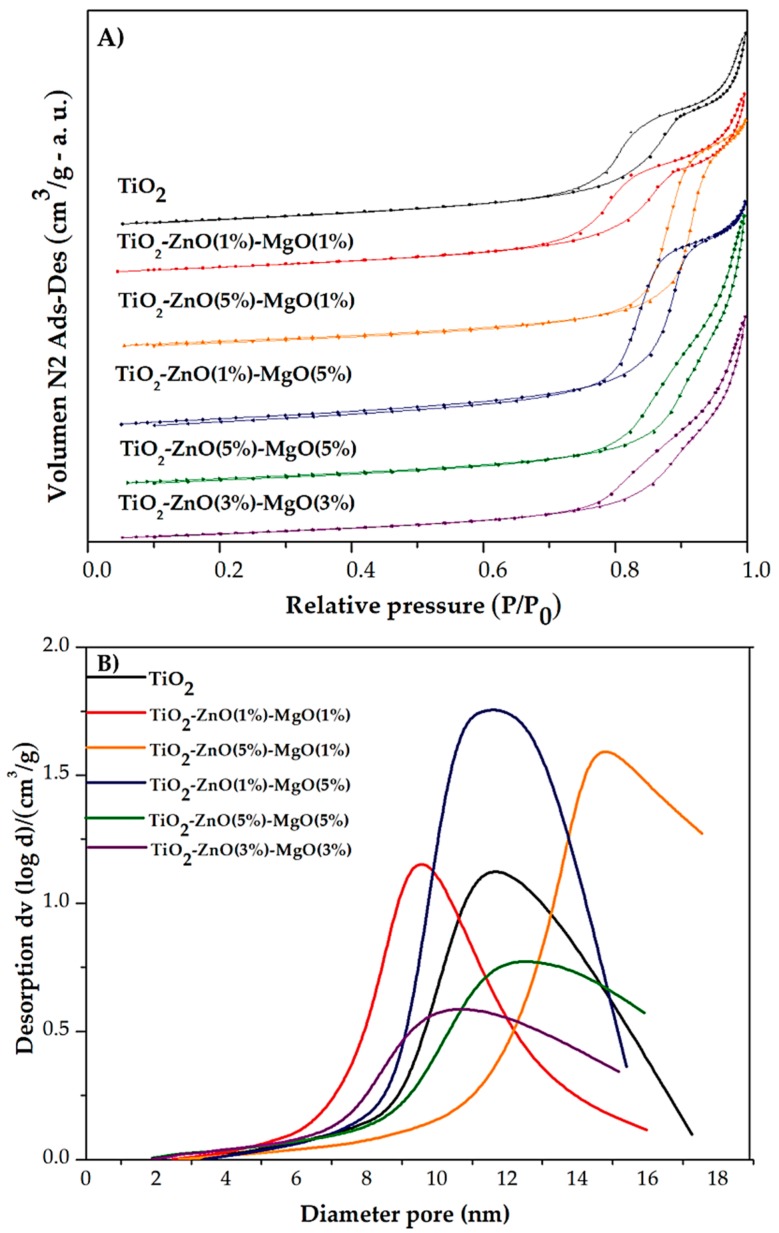
Nitrogen adsorption–desorption isotherms (**A**) and pore size distribution (**B**) of pure TiO_2_ and mixed oxide nanomaterials.

The N_2_ physisorption analysis was used to investigate the BET (Brunauer-Emmett-Teller) specific surface area (SSA), BHJ (Barret-Joyner-Halenda) pore volume, and average pore diameter of the pure TiO_2_ and MONs (TiO_2_-ZnO-MgO). Figure 3A shows the N_2_ adsorption-desorption isotherms of the pure TiO_2_ and MONs, and Figure 3B shows the pore size distribution. The isotherms of the materials (T1–T5), including pure TiO_2_, were identified as type IV, typical characteristics for mesoporous materials [23]. Additionally, the samples exhibited three different hysteresis types: H1-type for T3, H2-type for pure TiO_2_, T1 and T2, and H3-type for treatments T4 and T5. The hysteresis loop associated with isotherms is attributed to the capillary condensation of N_2_ gas occurring in the pores, which also confirms the presence of a mesoporous structure [23]. The change of hysteresis loop could be due to the existence of smaller or bigger pores in samples. Similar trends were previously reported by Deshmane et al. [24] and Amorós-Pérez et al. [25], when TiO_2_-supported metal (Cu, Co, Ni, Cr, Pd, Zn and Sn) was synthesized.

The textural properties of the synthesized materials are summarized in Table 2. Pure TiO_2_ showed a SSA of 61.53 m^2^/g, pore diameter of 9.97 nm and pore volume of 0.20 cm^3^/g. However, the SSA (57–71 m^2^/g), pore diameter (9.7–14 nm) and pore volume (0.18–0.31 cm^3^/g) of the MONs varied depending on the ZnO and MgO concentration (T1–T5) [26]. Comparable results were previously reported in pure TiO_2_ (58 m^2^/g) and/or doped-TiO_2_ with different transition metallic species, such as Cr (73 m^2^/g), Co (62 m^2^/g), Ni (53 m^2^/g) and Cu (63 m^2^/g) [25]. This might be attributed to the loading of metal oxides into mesoporous material, reducing their pore size and surface area by blocking the pores when the material is distributed on the particle’s inner surface [7,27].

### 3.3. X-Ray Diffraction

**Figure 4:**
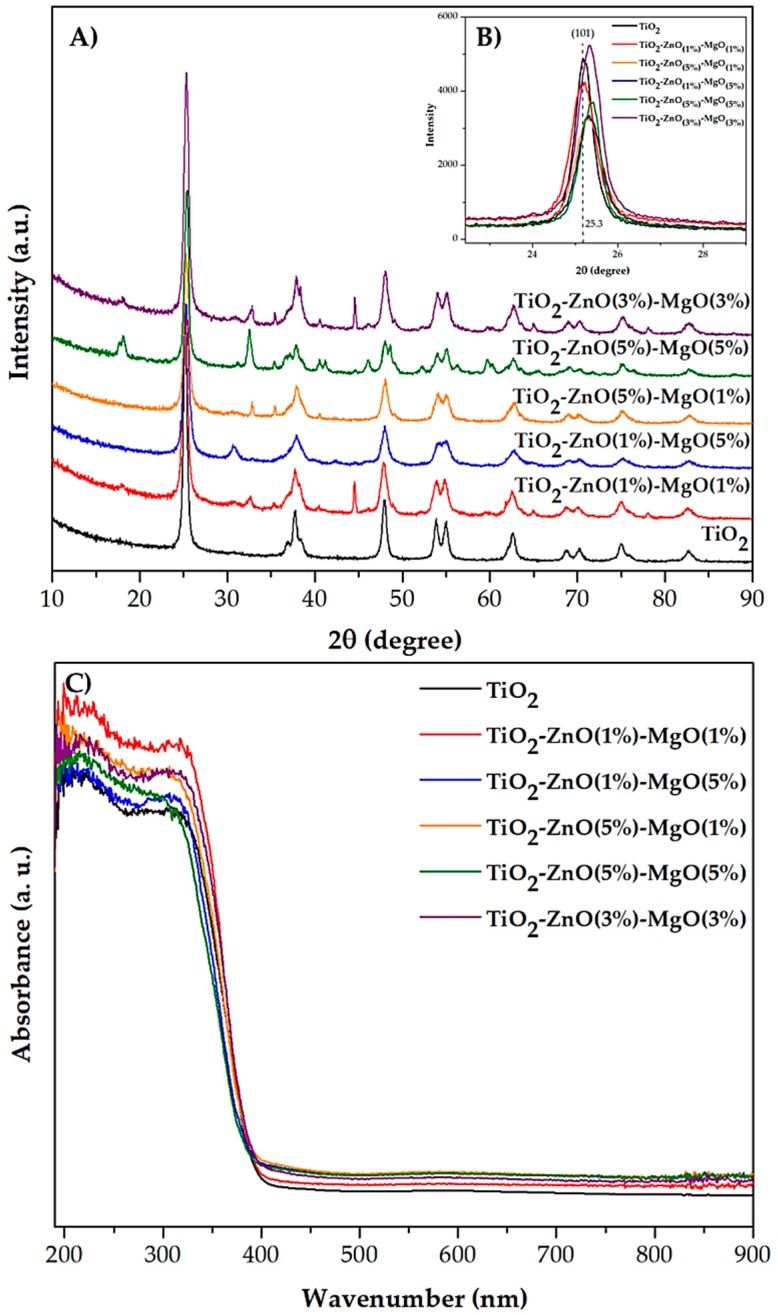
X-ray diffraction patterns (**A**,**B**) and UV-Vis (**C**) spectra of mixed oxide materials.

The crystalline structure of the pure TiO_2_ and MONs (TiO_2_-ZnO-MgO) was determined by X-ray diffraction (XRD) analysis. The diffractograms (Figure 4A) of the materials (T1 to T5) show the anatase phase of TiO_2_ corresponding to peaks at 2θ = 25.3°, 37.9°, 47.8°, 54.5°, 55°, 62.5°, 69°, 70°, 75° and 82°, with a respective Miller index of (101), (103), (200), (105), (211), (204), (116), (220), (215) and (303) planes (JCPDS 21–1272). In addition, the characteristic diffraction peaks of ZnO structures were observed at around 2θ = 31.7°, 34.5°, 36.3°, 47.5°, 56° and 62.7°, and were attributed to the (100), (002), (110), (102), (110) and (103) planes, respectively (JCPDS 36–1451). Similarly, diffractions at 36.7°, 42.71° and 62.0° were detected and attributed to MgO structure [28]. Nonetheless, the presence of ZnO and MgO did not promote significant changes in the anatase phase in the titania matrix [12]. However, in the amplification of stronger reflection (2θ = 25.3°, (101) plane) a notable shift in the nanomaterial in comparison with the reference TiO_2_ can be seen (Figure 4B). This was previously reported when Cu-doped TiO_2_ at 500 °C was synthesized [29]. The displacement of the (101) reflection was due to a slight modification in the anatase phase, promoting the incorporation of the dopant material into the titanium network. This small modification could promote a displacement in the whole diffractogram [30]. On the other hand, some treatments (TiO_2_-ZnO (3%)-MgO (3%) and TiO_2_-ZnO (5%)-MgO (5%)) showed reflection peaks around 18° (2θ), which are representative of Mg(OH)_2_ (JCPD 7–239). Some authors have suggested that an incomplete phase transformation of the precursor (magnesium di-ter-butoxide) to MgO during annealing can occur [28,31].

### 3.4. UV-Vis by Diffuse Reflectance

Figure 4C shows the diffuse reflectance spectra obtained for the MONs (TiO_2_-ZnO-MgO). All synthesized materials exhibited an absorption wavelength around 400 nm, which is characteristic of a TiO_2_ structure [32]. However, the optical absorption range (395.28–401.64 nm) of TiO_2_ changed in the presence of ZnO and MgO. Furthermore, the effect of the mixture of ZnO and MgO on the TiO_2_ *Eg* value is shown in Table 2. The *Eg* of synthetized nanomaterial (pure TiO_2_ = 3.13 eV) is influenced by the presence of Zn and Mg (3.09–3.14 eV) [29]. The differences in results may be due to the band gap of MgO (6–7.8 eV) being much wider than that exhibited by TiO_2_ (3.2 eV) and ZnO (3.37 eV) [27]. Similarly, Viswanatha et al. [33] observed a small shift in the absorption of ZnO (3.38 eV) by doping of Mg (3.34 eV). Furthermore, it has been reported that when two or more metals are combined, the optical properties of the MONs may be affected by an excess of doping material (≥10 wt.%) [7,30].

### 3.5. Infrared FTIR Analysis

**Figure 5:**
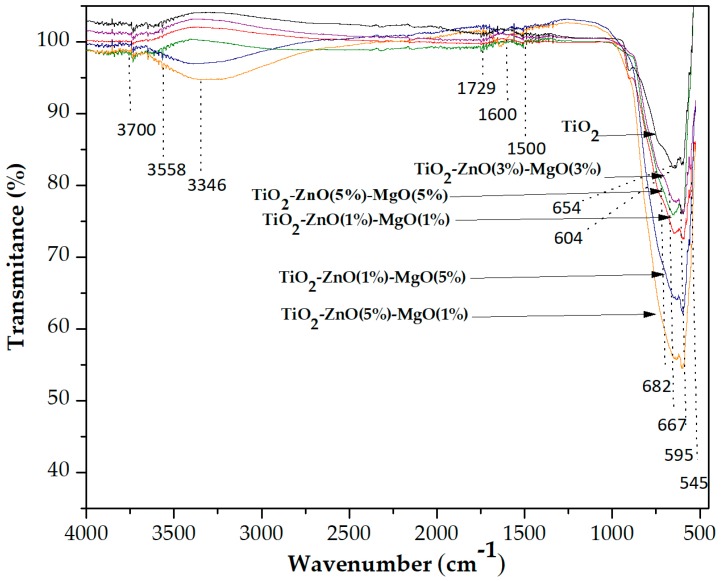
Fourier transform infrared spectra for TiO_2_ and mixed oxide nanomaterials.

Figure 5 shows the FTIR spectrum of the pure TiO_2_ and MONs recorded in the range of 4000 to 400 cm^−1^. Bands at 667, 654, 604 and 543 cm^−1^ were observed, and the vibrations in these regions are typical of the TiO_2_ anatase structure [32,34,35], and are related to the stretching mode of the Ti–OH, Ti–O and O–Ti–O bonds [8,11,33]. Also, a band around 545 cm^−1^ was detected in samples (T1–T5), attributed to the presence of the Zn–O bond [34]. Furthermore, a band at 667 cm^−1^ was detected, which confirmed the presence of MgO vibrations [16,33]. The absorption band around 1600 cm^−1^ was assigned to the O–H vibrations associated with physically adsorbed water in the samples [33]. The two bands at 1500 and at 1729 cm^−1^ can be associated with asymmetrical O=C–O vibrations and C=O stretching [35]. Also, the bands in the region around 1500 cm^−1^ may correspond to the Ti–O–C bond, which may originate from unreacted alkoxy groups (residual carbon after reaction) during the sol-gel process, indicating the interaction between the organic and inorganic components present in the precursor. This reaction could be promoted by the acidic conditions (pH = 3) of the medium [21,36]. Furthermore, bands ranging from 3346 cm^−1^ to 3700 cm^−1^ were detected, corresponding to the symmetric and asymmetric stretching of water molecules [32].

### 3.6. Color and Total Color Difference

Table 3 shows the color values (Luminosity (*L*), *a*, *b*, chrome and hue) and total color difference (TCD) of the pure TiO_2_ and MONs (TiO_2_-ZnO-MgO). Pure TiO_2_ had a white color (Luminosity (*L*) = 95.10, *a* = −0.41, *b* = 1.24, Chrome = 1.30, hue = 108.53). A statistical differences (*p* < 0.05) for all color parameters was observed in MONs (T1–T5) compared with pure TiO_2_. However, although the samples showed statistical differences, the MONs (T1–T5) had a similar white color to pure TiO_2_.

The color of TiO_2_-based materials is an important quality parameter in areas such as the food or pharmaceutical industries, since TiO_2_ is generally employed as a white pigment [10,11]. Differences in visual color can be classified by measuring the total color difference (TCD) as a very distinct (TCD > 3), subtle (1.5 < TCD < 3) or small difference (TCD < 1.5) [21]. Therefore, all the MONs presented a TCD < 3.0, and were thus classified as a subtle or small visual color difference. No significant changes in white color were reported when the CaCO_3_-TiO_2_ composite was prepared [11]. However, an evident visible change (dark green) in the color of the TiO_2_ powders was previously reported during the surface modification of TiO_2_ by Cr addition [29].

### 3.7. Antibacterial Activity

Table 4 shows the effect of MONs (100 μg/mL) on some pathogenic bacteria. Statistical differences (*p* < 0.05) were observed between treatments (T1–T5) compared with the drug control (ampicillin = 17–25 mm). Pure TiO_2_ showed a poor inhibition zone (5–9 mm) in all bacterial strain tested compared to the MONs (TiO_2_-ZnO-MgO) treatments. The MONs (T1–T5) showed significant antibacterial activities against *E. coli*, *S. paratyphi*, *S. aureus* and *L. monocytogenes*, but the antibacterial effect varied depending on the type of microorganism. The highest inhibition zone was observed for *E. coli* (16–18 mm), while the lowest was observed for *L. monocytogenes* (8–10 mm). The antibacterial effect of inorganic nanomaterials (TiO_2_, ZnO and MgO) is well documented, but studies have focused on the individual or bi-metallic combinations of these materials. However, research on the antibacterial effect of a ternary oxide material is limited.

Fu et al. [15] enhanced the antibacterial activity of nano-TiO_2_ in the presence of Au against *E. coli* and *Bacillus megaterium*. Jesline et al. [13] reported an inhibition zone of 14 and 17 mm against methicillin-resistant *S. aureus* using TiO_2_ and ZnO nanoparticles, respectively. However, the concentration of MONs to achieve a similar inhibition zone (T3 = 15 mm) against *S. aureus* was reduced by four times compared to those used by Jesline et al. [7]. Furthermore, the antibacterial effect of MONs on pathogenic bacteria studied could be ranked in the following order: *E. coli* > *S. paratyphi* > *S. aureus* > *L. monocytogenes* (Table 4). Li et al. [19] suggested that the antimicrobial effect of Ag–TiO_2_–Chitosan might be related to the species of bacteria. The authors reported a greater effect of the composite on *E. coli* (Gram-negative) than that of *Pseudomona aeruginosa* (Gram-positive), and mentioned that the differences in the antibacterial effect might be due to the variation in their cell wall composition. On the other hand, Lun et al. [35] reported that nano-texured TiO_2_ exhibited an equal level of lethality (>99%) on both Gram-negative and Gram-positive bacteria. 

The exact antibacterial mechanism of MONs is still unknown, but oxidative stress via the generation of reactive oxygen species (ROS) might cause lipid peroxidation of the cell wall, affecting the membrane fluidity and consequently disrupting cell integrity and promoting the release of the intracellular contents and death of the bacterial cells [36]. It is also believed that the accumulation of particles on the bacterial surface due to electrostatic forces could be another mechanism of the antibacterial effect of MONs [37]. These properties of MONs make them a promising antibacterial agent with great potential for industrial applications [12,37,38].

## 4. Conclusions

TiO_2_-ZnO-MgO mixed oxide (MONs)-based TiO_2_ was successfully synthesized using the sol-gel method. Textural properties such as the specific surface area, pore volume and pore diameter of the MONs increased or decreased depending on the ZnO and MgO concentrations, but preserved the anatase phase of TiO_2_ without perceptible changes in color. The MONs showed good antimicrobial activity against different Gram-negative and Gram-positive bacteria. Therefore, MONs have great antimicrobial potential for industrial applications.

## Figures and Tables

**Table 1:** Energy dispersive spectroscopy results of the composition (wt.%) obtained from the materials.

Sample	Code	Element			
		Ti	Zn	Mg	O
Undoped TiO_2_	-	44.90			55.10
TiO_2_-ZnO_(1 wt.%)_-MgO_(1 wt.%)_	T1	55.91	0.95	0.75	42.39
TiO_2_-ZnO_(5 wt.%)_-MgO_(1 wt.%)_	T2	59.38	5.02	0.90	34.70
TiO_2_-ZnO_(1 wt.%)_-MgO_(5 wt.%)_	T3	51.31	0.85	4.75	43.09
TiO_2_-ZnO_(5 wt.%)_-MgO_(5 wt.%)_	T4	68.72	5.70	5.29	20.28
TiO_2_-ZnO_(3 wt.%)_-MgO_(3 wt.%)_	T5	45.21	3.25	3.10	48.44

**Table 2:** Band gap energy (Eg) values and textural properties of pure TiO_2_ and mixed oxide (TiO_2_-ZnO-MgO) materials.

Treatment	Code	Eg (eV)	SSA (m^2^/g)	Pore Volume (cm^3^/g)	Pore Diameter (nm)
Undoped TiO_2_	-	3.13	61.53	0.20	9.97
TiO_2_-ZnO_(1 wt.%)_-MgO_(1 wt.%)_	T1	3.12	58.90	0.20	9.72
TiO_2_-ZnO_(5 wt.%)_-MgO_(1 wt.%)_	T2	3.14	57.52	0.27	14.78
TiO_2_-ZnO_(1 wt.%)_-MgO_(5 wt.%)_	T3	3.13	71.36	0.31	12.14
TiO_2_-ZnO_(5 wt.%)_-MgO_(5 wt.%)_	T4	3.14	54.85	0.21	11.82
TiO_2_-ZnO_(3 wt.%)_-MgO_(3 wt.%)_	T5	3.09	59.14	0.18	10.78

Eg = Band gap energy; SSA = specific surface area.

**Table 3:** Color values (Luminosity (*L*), *a*, *b*, chrome and hue) and total color difference (TCD) of pure TiO_2_ and mixed oxides (TiO_2_-ZnO-MgO) materials.

Treatment	Code	Luminosity	*a*	*b*	Chrome	hue	TCD
Undoped TiO_2_	-	95.10 ± 0.04 ^b^	−0.41 ± 0.01 ^b,c^	1.24 ± 0.03 ^b^	1.30 ± 0.02 ^a,b^	108.53 ± 0.40 ^c,d^	-
TiO_2_-ZnO_(1 wt.%)_-MgO_(1 wt.%)_	T1	94.63 ± 0.05 ^b^	−0.31 ± 0.02 ^a,b^	1.12 ± 0.06 ^c^	1.15 ± 0.05 ^b^	105.30 ± 1.57 ^d^	0.49
TiO_2_-ZnO_(5 wt.%)_-MgO_(1 wt.%)_	T2	96.73 ± 0.14 ^a^	−0.77 ± 0.04 ^c^	1.60 ± 0.03 ^a^	1.56 ± 0.33 ^a^	114.93 ± 0.99 ^c^	1.70
TiO_2_-ZnO_(1 wt.%)_-MgO_(5 wt.%)_	T3	86.21 ± 1.18 ^c^	−0.11 ± 0.02 ^a^	0.17 ± 0.06 ^e^	0.17 ± 0.02 ^d^	125.86 ± 1.56 ^b^	8.95
TiO_2_-ZnO_(5 wt.%)_-MgO_(5 wt.%)_	T4	85.84 ± 0.68 ^c^	−-0.40 ± 0.03 ^b^	0.48 ± 0.01 ^d^	0.59 ± 0.06 ^c^	134.00 ± 1.52 ^a^	9.29
TiO_2_-ZnO_(3 wt.%)_-MgO_(3 wt.%)_	T5	97.34 ± 0.09 ^a^	−0.62 ± 0.01 ^c^	1.37 ± 0.02 ^b^	1.52 ± 0.01 ^a,b^	115.90 ± 1.43 ^c^	2.25

Values are the average of triplicate determinations from three different experiments (n = 9) ± standard deviation (SD). Different lowercase letters in the same column indicate significant differences ((one-way ANOVA/Tukey test for all variables (*p* < 0.05), except for luminosity and *b* values, where Kruskal-Wallis/Multiple comparisons of mean ranks for all groups test were applied (*p* < 0.05)).

**Table 4:** Antimicrobial activity of pure TiO_2_ and mixed oxide (TiO_2_-ZnO-MgO) materials.

Treatment	Code	*E. coli* (mm)	*S. paratyphi* (mm)	*S. aureus* (mm)	*L. monocytogenes* (mm)
Ampicillin (C+)	-	22.33 ± 0.51 ^a^	25.66 ± 1.52 ^a^	18.33 ± 0.57 ^a^	17.33 ± 1.15 ^a^
Distilled water (C-)	-	0	0	0	0
Undoped TiO_2_	-	9.33 ± 0.57 ^c^	9.33 ± 0.57 ^c^	8.00 ± 1.00 ^e^	5.66 ± 0.57 ^d^
TiO_2_-ZnO_(1 wt.%)_-MgO_(1 wt.%)_	T1	14.05 ± 0.42 ^b^	16.00 ± 1.24 ^b^	12.33 ± 0.87 ^d^	8.83 ± 0.75 ^b,c^
TiO_2_-ZnO_(5 wt.%)_-MgO_(1 wt.%)_	T2	14.00 ± 0.89 ^b^	17.00 ± 1.26 ^b^	12.00 ± 0.94 ^d^	9.58 ± 0.66 ^b,c^
TiO_2_-ZnO_(1 wt.%)_-MgO_(5 wt.%)_	T3	14.91 ± 0.66 ^b^	18.33 ± 0.63 ^b^	15.16 ± 0.30 ^b,c^	10.00 ± 0.89 ^b,c^
TiO_2_-ZnO_(5 wt.%)_-MgO_(5 wt.%)_	T4	14.66 ± 0.51 ^b^	17.50 ± 1.04 ^b^	14.66 ± 0.81 ^b^	8.66 ± 0.81 ^b^
TiO_2_-ZnO_(3 wt.%)_-MgO_(3 wt.%)_	T5	14.76 ± 0.35 ^b^	17.76 ± 0.30 ^b^	13.83 ± 0.75 ^c^	8.33 ± 0.51 ^c^

Values are the average of triplicate determinations from three different experiments (n = 9) ± standard deviation (SD). Different lowercase letters in the same column indicate significant difference ((one way ANOVA/Tukey test for *S. aureus* and *L. monocytogenes* data were applied (*p* < 0.05); while a Kruskal-Wallis/Multiple comparisons of mean ranks for all groups test were applied on *E. coli* and *S. paratyphi* data (*p* < 0.05)).

## Supplementary Materials

The following are available online at https://www.mdpi.com/1996-1944/12/5/698/s1, Table S1: Results of normal distribution tests, using Levene’s and Shapiro-Wilk W tests (*p*-values) for variables studied.
