# Supplementary material for: Synthesis and Characterization of TiO2-ZnO-MgO Mixed Oxide and Their Antibacterial Activity
Source: Materials (Basel). 2019 Feb 27;12(5):698. doi: 10.3390/ma12050698 (PMC6427488; doi:10.3390/ma12050698)
Supplement: Supplementary file 1 [file materials-12-00698-s001.pdf]

Supplementary Materials

Synthesis and Characterization of TiO_2-ZnO-MgO Mixed Oxide and Their Antibacterial Activity

Running Title: Antibacterial Activity of Mixed Oxide Nanomaterials

Luis M. Anaya-Esparza [1], Efigenia Montalvo-González [1], Napoleón González-Silva [2], María D. Méndez-Robles [2], Rafael Romero-Toledo [3], Elhadi M. Yahia [4] and Alejandro Pérez-Larios [2,*]

1 Laboratorio Integral de Investigación en Alimentos, Tecnológico Nacional de México-Instituto Tecnológico de Tepic. Av., Tecnológico 255, Lagos del Country, 63175 Tepic, Nayarit, México; l_m_ae@hotmail.com (L.M.A.-E.); efimontalvo@gmail.com (E.M.-G.)

2 Universidad de Guadalajara, Centro Universitario de los Altos. División de Ciencias Agropecuarias e Ingenierías. Carretera a Yahualica Km. 7.5, 47600 Tepatitlán de Morelos, Jalisco, México; napoleon.gonzalez@cualtos.udg.mx (N.G.-S.); mdmendez@cualtos.udg.mx (M.D.M.-R.)

3 Universidad de Guanajuato, División de Ciencias Naturales y Exactas, Campus Guanajuato, Noria Alta S/N, Noria Alta, 36050 Guanajuato, Guanajuato, México; Ing_Romero2009@hotmail.com

4 Facultad de Ciencias Naturales, Universidad Autónoma de Querétaro. Avenida de las Ciencias S/N, Juriquilla, Santiago de Querétaro, 76230 Querétaro, México; yahia@uaq.mx

* Correspondence: alarios@cualtos.udg.mx

Table S1. Results of normal distribution tests, using Levene's and Shapiro-Wilk W tests (*p*-values) for variables studied.

Parameter	Variable	Levene's test (*p*-value)	Shapiro-Wilk W test (*p*-value)
Color parameters	Luminosity	0.0008	0.0057
	a	0.0992	0.4816
	b	0.0874	0.0194
	Chrome	0.1114	0.0736
	Hue	0.1540	0.2450
Antimicrobial activity	*Listeria monocytogenes*	0.4399	0.0839
	Staphylococcus aureus	0.2995	0.1029
	Escherichia coli	0.4541	0.0003
	Salmonella paratyphi	0.1908	0.0010
